# Evaluation of HIV-1 polymerase chain reaction (PCR) technique using dried blood spots (DBS) for diagnosis of perinatal transmission of HIV

**DOI:** 10.1186/1471-2334-12-S1-P46

**Published:** 2012-05-04

**Authors:** Saraswati Rajendran, John Kenneth, Anita Shet

**Affiliations:** 1 Chettinad Hospital and Research Institute, Chennai, India; 2 St John’s Research Institute, Bengaluru, India

## Background

Effective health care delivery to the majority of perinatally exposed infants is hampered by lack of access to accurate HIV diagnosis in infancy. Polymerase chain reaction is the most sensitive test to diagnose HIV-1 infection in children born to HIV seropositive mothers. The purpose of this study was to assess the feasibility and accuracy of using dried blood spot (DBS) technology in performing HIV-1 DNA PCR using Roche Amplicor HIV-1 DNA PCR version 1.5 for diagnosis in children less than 18 months of age.

## Materials and methods

This was a prospective observational study. 41 newborn infants of HIV-infected mothers were recruited and in addition 24 HIV-infected mothers, served as positive controls. DNA was extracted from filter paper using chelex resin and amplified using Roche Amplicor HIV-1 DNA PCR test. Sensitivity and specificity of the DBS PCR analysis was analyzed by comparing it with the results of PBMC’s (peripheral blood mononuclear cells).

## Results

Out of 66 DBS samples tested, 31 were positive, 35 negative and there was no indeterminate result. Whereas using PBMC, 32 samples were positive, 34 negative and 4 samples which were indeterminate initially were negative on repeat testing. All mothers were positive by both from DBS and PBMC’s. Overall sensitivity and specificity of DBS using Roche Amplicor DNA PCR was 97% and 100% respectively.

## Conclusion

PCR performed using DNA extracted from filter paper using chelex method is simple, sensitive and specific and can be used in resource limited settings.

